# Rapidly disseminating *bla*_OXA-232_ carrying *Klebsiella pneumoniae* belonging to ST231 in India: multiple and varied mobile genetic elements

**DOI:** 10.1186/s12866-019-1513-8

**Published:** 2019-06-24

**Authors:** Chaitra Shankar, Purva Mathur, Manigandan Venkatesan, Agila Kumari Pragasam, Shalini Anandan, Surbhi Khurana, Balaji Veeraraghavan

**Affiliations:** 1 Department of Clinical Microbiology, Christian Medical College, Vellore, Tamil Nadu India; 2 Department of Laboratory Medicine JRNA Trauma Centre, All India Institute of Medical Sciences, New Delhi, India

**Keywords:** *K. pneumoniae*, *bla*_OXA-232_, ST231, India, Insertion sequences, Transposons

## Abstract

**Background:**

Recently, in India, there has been a shift from NDM to OXA48-like carbapenemases. OXA-181 and OXA-232 are the frequently produced variants of OXA48-like carbapenemases. OXA48-like carbapenemases are also known to be carried on transposons such as Tn*1999*, Tn*1999.2* and it is also associated with IS1R carried on Tn*1999*. In India, there are no previous reports studying the association of mobile genetic elements (MGEs) with OXA48-like carbapenemases. The present study was aimed at determining the genetic backbone of OXA48-like carbapenemases to determine the role of MGEs in its transfer and to investigate the Inc plasmid type carrying *bla*_OXA48-like_.

**Results:**

A total of 49 carbapenem resistant *K. pneumoniae* which included 25 isolates from South India and 24 isolates from North India, were included in the study. Whole genome sequencing using Ion Torrent PGM was performed to study the isolates. OXA-232 was present in 35 isolates (71%). In 19 isolates (39%), *bla*_OXA48-like_ was associated with MGEs. Insertion sequences such as ISX4, IS1, IS3, IS*Kpn*1, IS*Kpn*26, IS*Kpn*25, IS*Spu*2, IS*Kox*1, IS*4321R*, IS*Ec*36, and IS*Pa*38; and transposons such as TnAs3 and Tn2, were present. Isolates from northern and southern India belonging to same sequence type (ST) had diverse genetic backbone for *bla*_OXA48-like_. ST14 isolates from north had IS5 and Tn3 families while from south they had IS1, IS5 and IS630 families. ST231 from north had IS5, IS6 and Tn3 families with *bla*_OXA-232_ while from south, IS1, IS3 and IS5 families were observed; with IS*Kpn*26 being present among isolates from both the regions. *bla*_OXA48-like_ was predominantly found on ColKP3 plasmid. ST231 was the predominant ST in 22 isolates (45%).

**Conclusion:**

OXA-232 is the predominant variant of OXA48-like carbapenemase with ST231 being the commonest ST of OXA48-like carbapenemase producing *K. pneumoniae* in India. Diverse MGEs have been associated with both *bla*_OXA-232_ and *bla*_OXA-181_ which contribute to their spread. The MGEs in the present study are different from those reported earlier. There is no clonal expansion of *bla*_OXA48-like_ producing *K. pneumoniae* since diverse STs were observed. Monitoring the genetic backbone of OXA48-like carbapenemase is essential to better understand the transmission dynamics of XDR *K. pneumoniae*.

## Background

OXA carbapenemases are oxacillinases which hydrolyse isoxazolylpenicillins. They have been divided into 12 groups based on amino acid sequences. OXA48-like is the commonly seen group among *K. pneumoniae*. OXA-181 and OXA-232 are the frequently produced variants of OXA48-like carbapenemases. OXA-181 and OXA-232 differ from each other by four amino acids: T104A; N110D; E168Q; S171A [1]. OXA-232 is a five amino acid variant of OXA-48 (T104A; N110D; E168Q; S171A; R214S). OXA-232 varies from OXA-181 by single amino acid (R214S) [1]. OXA-181 and OXA-232 have been reported with NDM-1 especially in India [2, 3]. Turkey, Morocco, Egypt, Libya and India are considered to be endemic for OXA48-like carbapenemases [4].

The *bla*_OXA48-like_ genes are always carried on plasmids. Initially, IncL plasmids mediated the spread of *bla*_OXA48-like_ genes. However, they have now been reported among other plasmid types such as IncH, IncA/C, IncX3 and ColKP3 [5–8]. OXA48-like carbapenemases are also known to be carried on transposons such as Tn*1999*, Tn*1999.2* and it is also flanked by IS1R carried on Tn*1999* [9, 10]. In India, there are no previous reports studying the association of mobile genetic elements with OXA48-like carbapenemases. Recently, in India, there has been a shift from NDM to OXA48-like carbapenemases [11]. Hence it is important to understand the role of mobile genetic elements (MGEs) in transfer of *bla*_OXA48-like_. The present study was aimed at determining the genetic backbone of OXA48-like carbapenemases in order to determine the role of MGEs in its transfer. The study also investigated the Inc plasmid type carrying *bla*_OXA48-like_.

## Methods

### Phenotypic characterisation

A total of 49 *K. pneumoniae* isolates which included 25 from Christian Medical College (CMC), Vellore, from South India, and 24 isolates from All India Institute of Medical Sciences (AIIMS), New Delhi, from North India, were included in the study. The isolates were identified by conventional biochemical methods as *K. pneumoniae* [12]. The antimicrobial susceptibility testing for imipenem (10 μg) and meropenem (10 μg) was performed for the isolates by Kirby Bauer disk diffusion method as recommended by Clinical and Laboratory Standards Institute (CLSI) and interpreted according to CLSI guidelines. *Escherichia coli* ATCC 25922 and *Pseudomonas aeruginosa* ATCC 27853 were used as the control strains for susceptibility testing. The isolates that were resistant to imipenem and meropenem as determined by CLSI guidelines were included in the study.

### Molecular characterisation

DNA was extracted from 18 to 24 h old cultures using Qiasymphony (Qiagen, Hilden, Germany) as per manufacturer’s instructions. Multiplex PCR for determination of carbapenemases such as *bla*_IMP_, *bla*_VIM_, *bla*_NDM_, *bla*_SPM_, *bla*_OXA48-like_ and *bla*_KPC_ were performed as described previously [2].

The isolates were subjected to whole genome sequencing using Ion Torrent PGM platform with 400 bp chemistry. Raw reads were assembled using Assembler SPAdes v.5.0 software in Torrent suite server version 4.4.3. The genome was annotated using RAST (Rapid Annotation using Subsystems Technology- http://rast.nmpdr.org/), Patric (Pathosystems Resource Integration Centre - https://www.patricbrc.org/) and the National Centre for Biotechnology Information Prokaryotic Genomes Automatic Annotation Pipeline (NCBI PGAAP) softwares. The resistance genes were identified using ResFinder version 2.1 (https://cge.cbs.dtu.dk/services/ResFinder/) and Multi-locus Sequence typing (MLST) was determined using database at https://cge.cbs.dtu.dk/services/MLST/ . Plasmids present in the genome were identified by PlasmidFinder version 1.3 available at https://cge.cbs.dtu.dk/services/PlasmidFinder/.

The presence of insertion sequences and other mobile genetic elements adjacent to *bla*_OXA-181_ and *bla*_OXA-232_ were determined by NCBI annotation and further using ISFinder (https://www-is.biotoul.fr/) to confirm the identity of insertion element.

Whole genome single nucleotide polymorphism (SNP) tree was constructed using CSI Phylogeny at https://cge.cbs.dtu.dk/services/CSIPhylogeny/ . For the phylogenetic tree, metadata was labelled using iTOL software at https://itol.embl.de .

## Results

The isolates from CMC, Vellore, were distributed over a span of 6 years: 2013 (*n* = 3), 2014 (*n* = 5), 2015 (*n* = 3), 2016 (*n* = 5), 2017 (*n* = 6) and 2018 (*n* = 3). All the isolates were resistant to aminoglycosides, β-lactams, fluoroquinolones and minocycline. Twenty one isolates were colistin resistant with minimum inhibitory concentration (MIC) ranging from 4 to 1024 μg/ml. All isolates except Kp21 and Kp22 were susceptible to tigecycline. The accession numbers for genomes, *bla*_OXA48-like_ variant, year of isolation, plasmid carrying *bla*_OXA48-like_ and MLST have been mentioned in Table 1. Among the CMC study isolates, 19 carried *bla*_OXA-232_ and six carried *bla*_OXA-181_. Three isolates co-expressed *bla*_NDM_ with *bla*_OXA48-like_ as mentioned in Table 1.

**Table 1:** Details of study isolates including accession numbers

Centre	Isolate no.	Accession no/ Bioproject ID	*bla*_OXA-48_ variant	Plasmid	Insertion sequence flanking *bla*_OXA-48_ variant	MLST
CMC, Vellore	Kp1	MPCT00000000	OXA-232	ColKP3	IS*Kpn*26, IS5 family; IS110 family	ST231
	Kp2	MOXL00000000	OXA-232	ColKP3/ IncFII	None	ST231
	Kp3	PUXB0000000	OXA-181 NDM-5	unidentified	None IS*Aba125*	ST147
	Kp4	MEBR00000000	OXA-232 NDM-1	unidentified	IS*Kpn2*6, IS5 family None	ST14
	Kp5	MDZG00000000	OXA-232	ColKP3	TnAs3, Tn3 family	ST231
	Kp6	MOXN00000000	OXA-232	ColKP3	ISX4, IS1 family; ISRaq1, IS3 family	ST231
	Kp7	MOXM00000000	OXA-232	ColKP3	None	ST14
	Kp8	MIEJ00000000	OXA-232	ColKP3	IS1A and IS1F, IS1 family	ST14
	Kp9	LZYN00000000	OXA-181	ColKP3	None	ST147
	Kp10	MCFO00000000	OXA-232	ColKP3	None	ST231
	Kp11	MCFP00000000	OXA-181	unidentified	None	ST43
	Kp12	NTHQ00000000	OXA-232	ColKP3	None	ST231
	Kp13	PJOP00000000	OXA-232	ColKP3	None	ST16
	Kp14	PKMV00000000	OXA-181	unidentified	None	ST147
	Kp15	PETC00000000	OXA-232	ColKP3	None	ST231
	Kp16	PKOL00000000	OXA-232	ColKP3	None	ST231
	Kp17	PKOK00000000	OXA-232	unidentified	IS*Kpn*26, IS5 family; IS*Spu*2, IS630 family	ST14
	Kp18	NSCV00000000	OXA-232	unidentified	None	ST231
	Kp19	NRSU00000000	OXA-232	ColKP3	None	ST231
	Kp20	PKOM00000000	OXA-181	IncA/C2	None	ST231
	Kp21	PPXS00000000	OXA-232	ColKP3	None	ST395
	Kp22	PPXT00000000	OXA-232	unidentified	IS*Kpn1*, IS3 family; IS4321R, IS110 family	ST570
	Kp23	PUIG00000000	OXA-181	unidentified	None	ST14
	Kp24	PYSM00000000	OXA-232	unidentified	None	ST231
	Kp25	PUIF00000000	OXA-232 NDM-5	ColKP3 unidentified	None IS*Aba125*	ST147
AIIMS Trauma Centre, New Delhi	Kp26	PWAF00000000	OXA-181	unidentified	IS*Kox*1, partial, IS66family	ST43
	Kp27	PWAD00000000	OXA-181	unidentified	IS*Kox*1, partial, IS6family	ST43
	Kp28	MNPB00000000	OXA-232	ColKP3	IS*Pa*38, Tn3 family; IS4321R, IS110 family	ST11
	Kp29	MNPC00000000	OXA-232	ColKP3	IS*Kpn*25, ISL3 family	ST11
	Kp30	MNPG00000000	OXA-232	ColKP3	IS*Pa*38, Tn3 family	ST11
	Kp31	MNPH00000000	OXA-232	unidentified	IS*Kpn*26, IS5 family	ST14
	Kp32	PRJNA494951	OXA-232	unidentified	TnAs3, Tn3 family	ST14
	Kp33	PRJNA494951	OXA-232	ColKP3	Tn2, Tn3 family	ST2040
	Kp34	MNPA00000000	OXA-232	unidentified	IS26, IS6 family; IS903, IS5 family; ISPa38, Tn3 family	ST231
	Kp35	PYUL00000000	OXA-181	unidentified	IS*Kox*1 IS66 family; IS*Ec*36 IS3 family; IS*Kpn*42 IS110 family	ST43
	Kp36	PRJNA494951	OXA-181	unidentified	IS*Kpn*1, IS3 family	ST43
	Kp37	PRJNA494951	OXA-181	unidentified	IS*Kpn*1 partial, IS3 family	ST11
	Kp38	PRJNA494951	OXA-232	unidentified	IS*Kpn*1, partial, IS3 family; IS5075, IS110 family	ST11
	Kp39	PWAH00000000	OXA-232	unidentified	None	ST101
	Kp40	PWAE00000000	OXA-232	ColKP3	None	ST231
	Kp41	PRJNA494951	OXA-232	ColKP3	None	ST231
	Kp42	PRJNA494951	OXA-181	ColKP3	None	ST16
	Kp43	PRJNA494951	OXA-181	IncA/C2	None	ST231
	Kp44	PRJNA494951	OXA-232	ColKP3	None	ST15
	Kp45	PRJNA494951	OXA-232	ColKP3	None	ST15
	Kp46	PRJNA494951	OXA-181	ColKP3	None	ST15
	Kp47	PRJNA494951	OXA-232	ColKP3	None	ST231
	Kp48	PRJNA494951	OXA-232	ColKP3	None	ST231
	Kp49	PRJNA494951	OXA-232	ColKP3	None	ST231

**Fig. 1:**
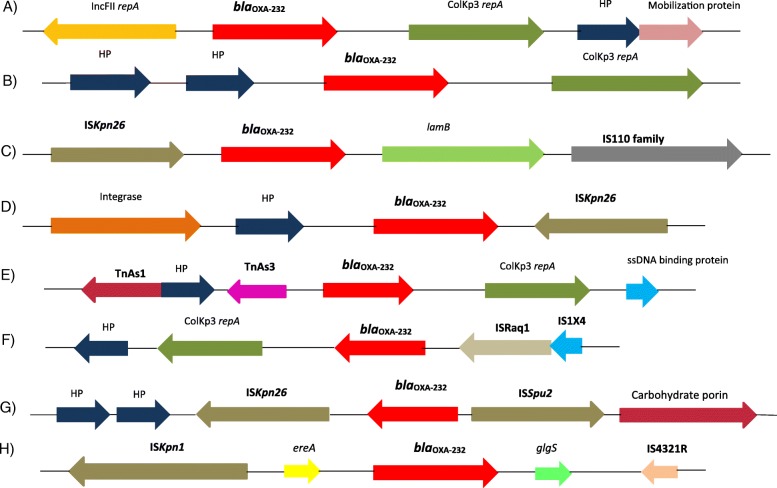
Mobile genetic elements flanking *bla*_OXA-48 like_ among *K. pneumoniae* from CMC, Vellore. **a** and **b**: *bla*_OXA-48 like_ without insertion sequences in Kp2 and Kp10 respectively. **c** to **h**: *bla*_OXA-48 like_ associated with insertion sequences and transposon in Kp1, Kp4, Kp5, Kp6, Kp17, KP22 respectively

In six isolates from CMC, *bla*_OXA-232_ was associated with insertion sequences as depicted on Fig. 1. Figure 1 also shows the genetic backbone among two isolates in which *bla*_OXA232_ is not flanked by insertion sequences. The genetic backbone is diverse among the isolates as shown in Fig. 1 even among isolates belonging to same sequence type. Isolates belonging to ST14 had insertions from IS1, IS5 and IS630 families while those of ST231 had insertions belonging to IS5, IS1, IS3 and Tn3 families (Table 1).

Seven sequence types were observed among the South Indian isolates which include ST231 (*n* = 12), ST14 (*n* = 5), ST147 (*n* = 4), ST16 (n = 1), ST43 (n = 1), ST395 (n = 1) and ST570 (n = 1). ST231 has been isolated throughout the study period. ST231 and ST43 belong to the same clonal complex (CC), CC43. ST231 is a triple locus variant of ST43 varying in *pgi, phoE* and *tonB* genes with 11SNPs.

**Fig. 2:**
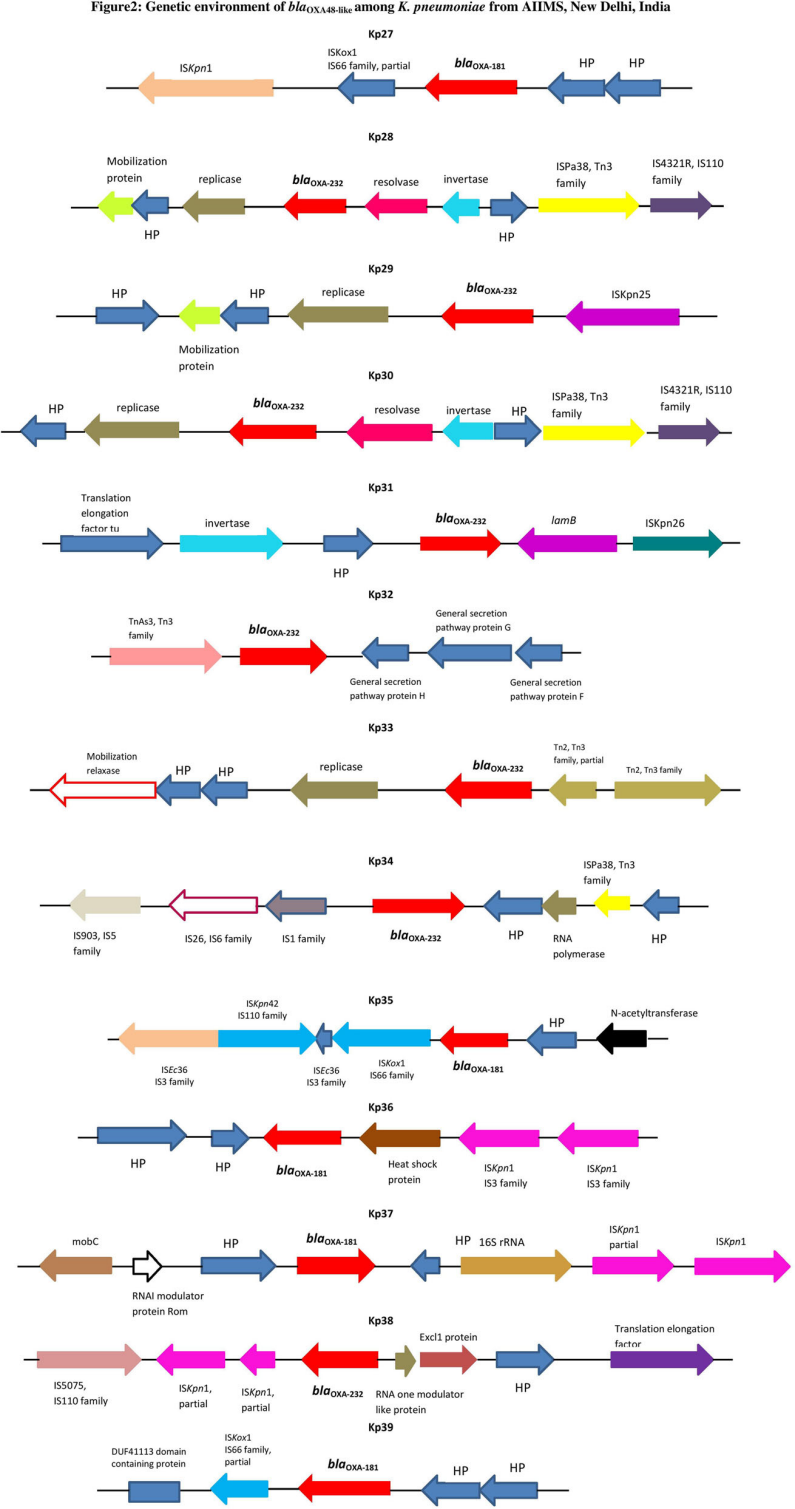
Genetic environment of *bla*_OXA48-like_ among *K. pneumoniae* from AIIMS, New Delhi, India Kp27, Kp28, Kp29, Kp30, Kp31, Kp32, Kp33, Kp34, Kp35, Kp36, Kp37, Kp38, Kp39

The isolates from AIIMS, New Delhi, were obtained during 2016 and 2017. The isolates belonged to diverse sequence types including ST231 (*n* = 7), ST11 (n = 5), ST43 (n = 4), ST14 (*n* = 2), ST15 (*n* = 3), ST16 (n = 1), ST101 (n = 1), and ST2040 (n = 1). CC11 including ST11, ST14, ST15 and ST2040, was predominant in north India. ST231 is predominantly present in both the study centres. Among the 24 isolates from AIIMS, eight were OXA-181 producers and 16 were OXA-232 producers. The genetic backbone among these isolates from New Delhi seems to be very diverse despite the clonality. Genetic backbone of isolates with *bla*_OXA48-like_ associated with mobile genetic elements is shown in Fig. 2.

As seen from Table 1, isolates from northern and southern India belonging to same clone had diverse genetic backbone for *bla*_OXA48-like_. Isolates from North belonging to ST14 had MGEs from IS5 and Tn3 families while from South they had MGEs from IS1, IS5 and IS630 families. A single isolate of ST231 from north had MGEs from IS5, IS6 and Tn3 families with *bla*_OXA-232_ while from south, IS1, IS3 and IS5 families were observed. This shows that there is no clonal expansion of OXA48-like producers in India.

Diverse mobile genetic elements have been associated with both *bla*_OXA-232_ and *bla*_OXA-181_ belonging to *bla*_OXA48-like_. This includes: a) insertion sequences such as ISX4, IS1, IS3, IS*Kpn*1, IS*Kpn*26, IS*Kpn*25, IS*Spu*2, IS*Kox*1, IS*4321R*, IS*Ec*36, and IS*Pa*38; b) transposons such as TnAs3 and Tn2, belonging to Tn3 family. IS*Kpn*26 has been seen among isolates from Vellore and New Delhi. This indicates the role of diverse MGEs in transmission of OXA48-like carbapenemases in India.

**Fig. 3:**
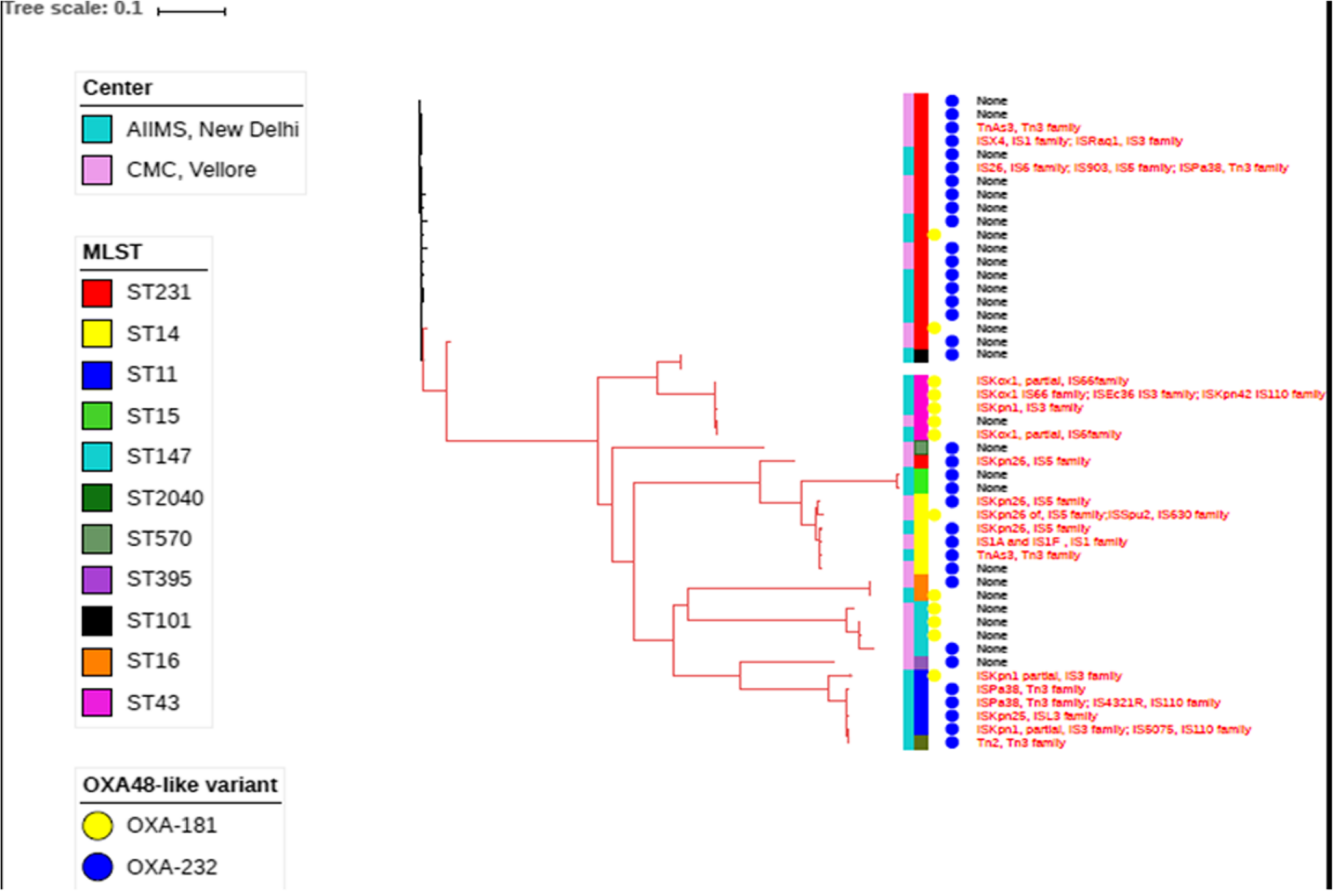
Whole genomes SNP based phylogenetic tree of the study isolates

Figure 3 shows the phylogenetic tree of OXA48-like carbapenemase producing *K. pneumoniae*. MLST, variant of OXA48-like carbapenemase and centre from where the isolates were obtained are shown in Fig. 3. Mobile genetic elements associated with OXA48-like has also been indicated.

## Discussion

The commonest variants of *bla*_OXA48-like_ reported among *K. pneumoniae* are *bla*_OXA-181_ and *bla*_OXA-232_. In the present study, significantly, 80% of the isolates were *bla*_OXA-232_ producers. In 14 of the study isolates, *bla*_OXA-232_ was associated with mobile genetic elements such as insertion sequences (IS) and transposons. Interestingly, among the isolates with IS, the regions flanking *bla*_OXA-232_ were diverse. No two isolates had the same genetic environment even among the isolates in which *bla*_OXA-232_ was not flanked by IS. IS*Kpn26* was found with *bla*_OXA-232_ in four isolates.

Tn*1999* and its isoforms have been frequently described carrying *bla*_OXA-232_ along with IS1R [9, 10, 13]. IS*Ecp*1 was reported among isolates from France and Brunei belonging to ST14 and ST231 [14, 15]. However, in the present study these mobile genetic elements were absent and significantly different from global isolates. Also, IncL/M type of plasmids are frequently found carrying *bla*_OXA48-like_ gene [16]. However, in the present study, none of the isolates harboured IncL/M plasmid. In contrast, in most of the isolates *bla*_OXA48-like_ gene was present on ColKP3 plasmid and on IncA/C2 in one of the isolates. IncA/C harbouring *bla*_OXA48-like_ gene has been previously reported [7]. A recent study in the US reported *bla*_OXA-232_ in all the study isolates to be present on ColKP3 plasmid [17].

In two of the study isolates, along with *bla*_OXA48-like_, *bla*_NDM-5_ was also present. *bla*_NDM-5_ was flanked by IS*Aba125* which is frequently associated with *bla*_NDM_ [18, 19]. Both these isolates were of ST147 isolated during 2013 and 2018. *bla*_OXA-181_ and *bla*_NDM-5_ has been previously reported in USA and South Korea [17, 20]. Similar to the present study, coexistence of *bla*_OXA-181_ and *bla*_NDM-5_ have been reported among *E. coli* and *K. pneumoniae* [20, 21].

Totally, 11 sequence types were observed in the present study. These were diverse and the two major clonal complexes were CC11 and CC43. ST14 and ST147 have been frequently reported among OXA48-like producing *K. pneumoniae* in various regions such as North America and Germany [22, 23]. ST14 and ST147 have been described as international high risk clones associated with extensively drug resistant (XDR) *K. pneumoniae* [24]. ST395 has also been reported among European and African OXA48-like producing *K. pneumoniae* [15].

## Conclusion

OXA-232 is the predominant variant of OXA48-like carbapenemase with ST231 being the commonest ST of OXA48-like carbapenemase producing *K. pneumoniae* in India. Diverse MGEs have been associated with both *bla*_OXA-232_ and *bla*_OXA-181_ which contribute to their spread. The MGEs in the present study are different from those reported earlier. There is no clonal expansion of *bla*_OXA48-like_ producing *K. pneumoniae* since diverse STs were observed. Among isolates belonging to same ST, diverse MGEs were observed associated with *bla*_OXA48-like_. Monitoring the genetic backbone of OXA48-like carbapenemase is essential to better understand the transmission dynamics of XDR *K. pneumoniae*.

## Data Availability

The datasets used and analysed during the current study are available from the corresponding author on reasonable request. The whole genome sequences are deposited in GenBank with accession numbers provided in Table 1 of the manuscript.

## Abbreviations

ATCC: American Type Culture Collection
CC: Clonal Complex
CLSI: Clinical and Laboratory Standards Institute
Inc.: Incompatibility
IS: Insertion sequence
MGE: Mobile Genetic Elements
MLST: Multi-locus sequence typing
NCBI: National Centre for Biotechnology Information
NDM: New Delhi metallo-β-lactamase
OXA: Oxacillinase
Patric: Pathosystems Resource Integration Centre
PCR: Polymerase Chain Reaction
PGAAP: Prokaryotic Genomes Automatic Annotation Pipeline
RAST: Rapid Annotation using Subsystems Technology
SNP: Single Nucleotide Polymorphism
ST: Sequence Type
Tn: Transposon
XDR: Extensively Drug Resistant
